# The Malaysian Food Barometer Open Database: An Invitation to Study the Modernization of Malaysian Food Patterns and Its Economic and Health Consequences

**DOI:** 10.3389/fnut.2021.800317

**Published:** 2022-01-19

**Authors:** Jean-Pierre Poulain, Laurence Tibère, Elise Mognard, Cyrille Laporte, Tristan Fournier, Ismail Mohd Noor, Anindita Dasgupta, Yasmine Alem, Kremlasen Naidoo, Anne Dupuy, Amandine Rochedy, Pradeep Kumar Nair, Neethianhantan Ari Ragavan

**Affiliations:** ^1^Centre d'Études et de Recherche: Travail, Organisation, Pouvoir (CERTOP) UMR CNRS 5044, Université de Toulouse, Toulouse, France; ^2^Chair ≪ Food Studies: Food, Cultures & Health ≫, Université de Toulouse, Toulouse, France; ^3^Faculty of Social Sciences and Leisure Management, University Malaysia, Subang Jaya, Malaysia; ^4^Center for Asian Modernisation Studies (CAMS), Taylor's University, Subang Jaya, Malaysia; ^5^Institut de Recherche Interdisciplinaire sur les Enjeux Sociaux (IRIS), Centre National de la Recherche Scientifique (CNRS), Paris, France; ^6^Faculty of Social Sciences and Leisure Management, Taylor's University, Subang Jaya, Malaysia

**Keywords:** open science, sociology of food, health, nutrition, meals norms, food practices, food transition, compacted modernization

## Introduction

The study was conducted within the framework of the chair of “Food Studies: Food, Cultures and Health” created jointly by Taylor's University (Malaysia) and the University of Toulouse Jean Jaurès (France). The Malaysian Food Barometer (MFB) is a recurrent survey focusing on the transformations of food patterns of the Malaysian population. MFB works with national representative samples from a socio-anthropological theoretical standpoint.

Since the 1990s, two major nutrition surveys namely the first Malaysian Adult Nutrition Survey MANS 2003 (1), which was repeated in 2014. However, these studies focus on individual food consumption, assessed in terms of nutritional composition of the diet, including some basic sociodemographical characteristics. MFB is a complementary survey to MANS (2003 and 2014) that explore in depth the sociological and ethnological dimensions of Malaysian food habits. The MFB collects face-to-face data on food-related practices including social norms, attitudes, cultural representations, and routines and their supposed sociocultural and demographic determinants. The focus includes the repartition of food practices at home and away from home, sources of food, food socialization of the meals and food intakes, food temporality, and perception of risks in food. A series of indexes were assigned to individuals within the phenomena of transition (demographic, nutrition, food, and protein) and of “compacted modernization” experienced by some Asian countries (2–5).

The theoretical objective of the project consists of studying the social, ethnic, and cultural diversification of food patterns of Malaysians and its changes in the context of modernization. The applied objective of the research is to analyze: first the impact of these transformations on the technical and economic organization of the food industry and service sector and second the consequences of changes in food consumption in terms of public health, especially on non-communicable diseases.

## Malaysian Society

The study of Malaysian eating patterns is a scientific challenge due to the multicultural character of the society coupled with the fact that it is facing a very rapid modernization. This phenomenon that we can call “compacted” modernization (6, 7) that impacts the ways to eat and the values system attached to food in a given population (8). Compacted modernity is characterized by some sociodemographic transformations, linked to the demographic transition like rapid urbanization, decrease of fertility, and reduction in the size of the household including some socioeconomic changes like increase in purchasing power and emergence of a middle class (9, 10). The epidemiological transition can be referred to as changes and the causes of mortality from epidemic diseases to cardiovascular diseases, cancers, and degenerative diseases (11–13). All these structural transformations have affected the lifestyles and the food habits of the various ethnic groups, which make the Malaysian population (14). Many countries, including Malaysia, are facing obesity epidemic that raises concerns about the negative effects on health that has stimulated researchers to focus on the food cultures and lifestyle transformations leading to the development of this issue.

Although most of the above characteristics can be found in modern countries, Malaysian social context has in addition two distinct characteristics. The first one is its multi-ethnicity character. Officially Malaysian society consists of three main ethnic groups, i.e., Malay, Chinese, and Indian (and a few minorities groups). Each group has its own food culture with its emblematic dishes, its taboos, and restrictions, its eating rituals, its meal structures, and its symbolic dimensions of food. However, “ethnic” categories are not homogeneous in Malaysia. For example, Indians may belong to different religions: Hindus, Muslims, Sikhs, Buddhists, Christians, or members of New Religious Movements. They can also speak different languages like Bahasa Malaysia, English, and Indian languages like Tamil, Hindi, Urdu, Malayalam, etc. In addition, they may be identified by a caste, by region of origin, etc. and may live in Malaysia for several generations or just arrived. Similarly, for the “Chinese” people, they may be Buddhist, Taoist, Christian, Muslim, or free thinker. They may speak Hakka, Hokkien, Cantonese, Teochew, and Mandarin. They may have several ethnic belonging, Hakka, Cantonese, and Wu. Furthermore, there are Malaysians in the official “Others” categories, such as the non-Malay Bumiputra, Dusun, Iban, and Kadazan. We must add foreigners living in Malaysia to this heterogeneous group, including expats (executives and domestic helpers), and international retirees on the “second home” program. But boundaries between the three main groups are not totally hermetic. There is even a certain “porosity” resulting from overlapping religious affiliation and language competence and interpersonal relationships across ethnic boundaries and through friendship. This “porosity” results also from the usage behind the primary “race” identity, religious conversion, and “crossbreeding” (multi-ethnic people) from historical institutionalized mixed marriages (e.g., in the Baba-Nyonya community).

The second characteristic is the high frequency of food consumed outside home, which is one among the highest rates in world. Historically, eating out-of-home, especially street food, is an important tradition in Asian countries. With the increase of urbanization, the opportunities for Malaysians to eat out have increased greatly, and the costs are sometimes cheaper than the home-made meals.

The MFB is a tool to identify and study the sociocultural determinants of the Malaysian food habits. It focuses simultaneously on the practices and on the representations of food cultures. The aim of MFB is to help understand the food life styles and the different food contexts of the various Malaysian ethnic groups and “middle class” in order to elucidate their process of making food decisions.

## Theoretical Framework

**Figure 1:**
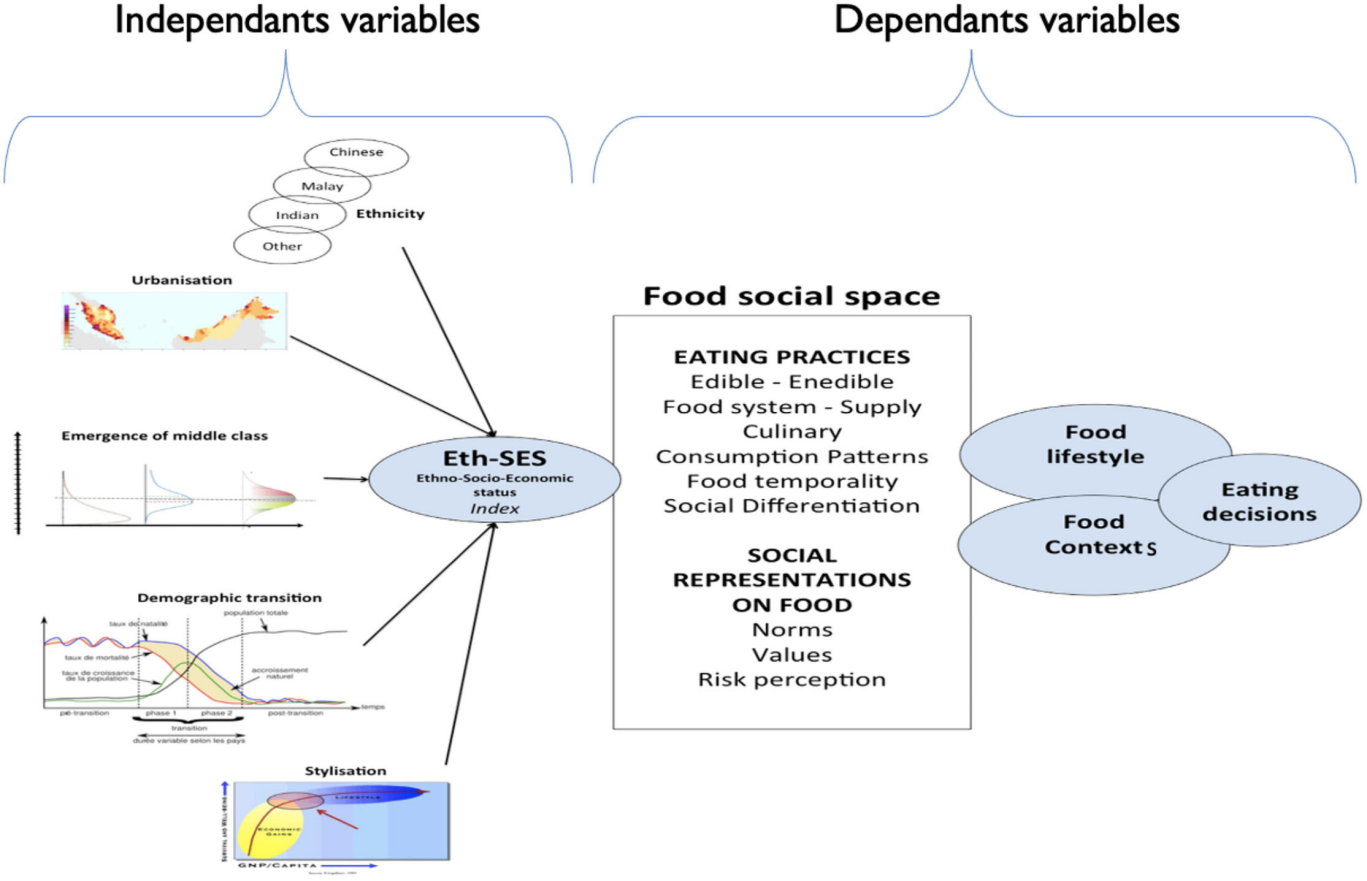
Malaysian Food Barometer (MFB) conceptual framework.

The theoretical framework of this database (see Figure 1) breaks with the dominant reading grid in nutrition, based on theories of rational choice or programmed action. The adopted theoretical stand postulates that a significant part of eating behavior is “unthoughtful,” and that dietary decisions are embodied in behavioral scenario predefined by societies and cultures. It is what we use to call according to a long anthropological tradition “pattern.” Thus, social influences can come from: (1) social positions of eaters (ethnicity, income, education, job, marital status, etc.), (2) food patterns defined by cultures (meals structures, routines, and eating contexts), and (3) social interactions with people who prepare the food and with whom the meals are taken and shared. The database, therefore, explores blind spots of traditional nutritional surveys. It opens a dialog with the nutritional sciences. “Food patterns” are “used” by eaters, and by the household members, who shop, cook, and prepare meals. They are used also by all those who directly or indirectly participate in the production, processing, and distribution of food products and services. They constitute the cognitive infrastructure, which allows these actors to coordinate and contribute to the functioning of the food system, its social “orchestration” (15).

On the question of decision, there are rooms for decision-making, but they are embedded in the structures and categories of food systems. Food models are therefore the infrastructure on which decisions are made. Eaters use these patterns like if they were “natural,” and therefore they have little awareness of their existence (16, 17).

Thus, this theoretical framework stands at a distance from the classic nutritional approach, explicitly included in the theories of rational choice. Therefore, decisions of the eaters are nested in culturally defined action scenarios. There are some decisions, but they take place in a socially determined room of freedom. Then the theoretical framework considers: (1) the multicultural characteristics of Malaysian society, (2) the transformations of this society under the influence of rapid modernization (“compacted modernization”), (3) the consequences of this modernization on “food social space,” both on food representations and on behavior patterns, and (4) contextualized decisions.

## Methodology

The MFB is a recurrent survey that studies the socioeconomic, demographic, and cultural determinants of food consumption. It focuses on the transformations of food practices and representations. It studies the mutations of food habits and tries to identify their possible influences on health, especially on non-transmissible diseases in which food is involved. It complements traditional nutritional surveys and aims to participate in the development of prevention programs (5). MFB is based on a mixed methodology developed at the national level. The qualitative approach precedes and prepares basis for the quantitative survey. The method is based on the distinction between social norms and practices. This approach has been developed over the last three decades in France and used in several national surveys, including the Barometer “Santé Nutrition” of the National Institute for Prevention and Health Education (INPES).

The data on eating habits are based on a “24-h recall” approach. The interviewees were asked to list all foods and drinks intake of the day before, both during and between main meals. It is quite a common method used to assess individuals food intake mainly used in nutrition science surveys, but we have adapted and modified it taking into account the sociocultural dimensions we wish to investigate. In the early 1980s, sociologists and economists were engaged in intense research activity focusing on food consumption surveys. The results of their work generated some theoretical debates, which gave rise to methodological advances that subsequently benefitted our approach to the current investigation. These include awareness of the need to consider the status of the variables and the data collection techniques.

For the quantitative research on eating practices, we encountered an obstacle related to the use of declarative methods to uncover, or at least get data as close as possible to the actual behavior of individuals. For example, when we ask individuals to describe the meals they ate the day before and if they have not eaten “as usual,” or if they ate differently from their usual pattern, “what they think they should have done,” they feel uncomfortable. Indeed, what should their answer be? What they actually did, or what they usually do?

The problem, methodologically, is that all individuals do not solve this dilemma in the same way. Some of them, respecting the instructions of the interviewer, faithfully describe the food intake of the day before, whereas others, eager to report their usual way of eating, are tempted to change their statement from the actual to the usual, to reduce the cognitive dissonance they feel. All seek to translate what they think is the reality of their food practices. In the second case, the data collected can be said to be more related to their perception of “social norms,” which are a mix of social and nutritional requirements than to their actual practices. Thus, the data obtained have a fairly weak empirical value because it represents neither a complete picture of the real behavior of individuals, nor of the social representations (norms and values) relating to food in the social group being studied. In an attempt to resolve this ambiguity, some studies have developed and accommodated the development of a collection method, which facilitates the distinction between practices and norms, using a questionnaire administered during a face-to-face interview (18, 19). This is done by first inviting people to say what they consider to be a “proper meal,” a “proper breakfast,” a “proper lunch,” etc. This is presented to them as taking place in an ideal setting when nothing has disturbed the material organization of the preparation and consumption of these meals. This method is an extension of the work of Mary Douglas (20) on “deciphering a meal.” Through this process, the social norms are collected for the meals under consideration. In the second step, when the interviewee is “liberated” from the normative pressures by his or her statement, another series of questions is proposed to help the individual to rebuild his or her food day. The interviewer begins by specifying that what now interests the research team is what really happened, what has really been eaten. The enumerator explains that, working at the level of the total population, it is not a problem if the meals eaten by the interviewee differ from what has been said in the first part of the questionnaire, when the informant tells what she or he thinks should be done, or what she or he usually does.

The first type of data corresponds to social norms, that is to say, provides an aggregate of guidelines that are rooted in cultural, social, and family traditions. They result from the specific socialization of an individual. But these norms are also impacted by the prevailing discourses of public health in relation to diet, or by the pressure of prevailing models of desirable body shape. The second type of data always retains the status of declarative data but is much closer to the actual practices of individuals. With such a method, the data collected gained precision, and it is possible to distinguish norms and practices and their relationships with each other, particularly for the exploration of various forms of change. The improvement of data collection methods is an important issue in this research. The ability to distinguish between norms and practices allows a deeper understanding of the transformation of eating habits. The distinction between norms and practices is a solution devised to improve the empirical quality of the data, but it has a cost because it greatly increases the length of the questionnaire and almost always requires face-to-face data collection.

### Questionnaire and Variables

The MFB studies the social, ethnic, and cultural diversification of food habits in Malaysia. It studies the evolution of food consumption, both at home and outside the home and identifies the consequences in terms of market factors and public health. It was based on a national representative sample of 2,000 respondents of 15 years old and above. The sampling methodology is a semi-randomized approach, based on the regions within Malaysia and their degree of urbanization (21) than a quota system based on age and ethnicity was also applied.

The questionnaire (see Annex 1) has 6 main parts: sociodemographics and ethnicity indicators, food norms, food intake in the last 24 h (recall), cooking practices, social representations of food, health and risk issues. It comprises 66 items and more than 1,400 variables and 58 closed and multiple-choice questions, consisting of standard questions used in sociology to describe the sociodemographics of a population (22), and questions that have been used in prior studies (2, 18, 23–25). The questionnaire was translated in three languages (English, Malay, and Chinese), and retro-translated to ensure the right meaning of the questions.

A complete presentation of the variables is available in the report (2). Here, we would like to highlight three essential points: the variables dedicated to ethnicity, the variables relating to eating habits, and some indexes of social position.

1. For ethnicity, data are not just what “is written on identity cards,” but include:
  - Assigned ethnicity.
  - Self-declaration.
  - Declaration of interviewee for the ascendants and spouse and spouse ascendants.
  - Hierarchy by the individual of ethnicity, religion, and citizenship.
2. Indexes of social position:
  - The “Modernization index” is a weighted combination of level of urbanization, size of household, level of income, evolution of income, level of education. Modernization index = (Urb × 3) + (Size household × 2) + (Income × 3) + (Evolution income × 1) + (Education × 2).
  - The “Ethnicity index” is a weighted combination of assigned group (Malay, Chinese, Indian, and others), self- declaration, auto-definition, self-declaration of the family (spouse, parents and grandparents of interviewee and spouse), religion, and intensity of religion practice.
  - EthSocPos categories combine “Assigned ethnicity” and “Income available per person” 12 categories are created:
  - 4 ethnicities (non-Malay Bumiputra, Malay, Indian, and Chinese).
  - 3 income categories (20% lowest, 60% middle, and 20% highest).
  Finally, it allows to a multicriteria approach of ethnic affiliations and social positions to analyze and sort out the influences of determinations of ethno-cultural origin and those linked to social positions.
3. Variables relating to the levels of the “act of eating.” The collection of food consumption data considers:
  - Sociocultural representations relating to food and eating practices.
  - Social norms defining meals.
  - Food practices of the day before the survey reconstructed using a 24-h reminder (adapted to avoid injunctions from Western categories, etc.).
  - Social contexts of consumption (places and people who are sharing the intake).
  - Social interactions.
  - Distribution of food intakes between home and out of home for the days before and the week (7 days) preceding the survey.

## Data Collection

The data collection was done face to face by enumerator trained by the research team. The objective was to help them to understand the key points of the methodology and the use of the questionnaire. The training of enumerators included role plays during which they alternated from the position of interviewer to that of interviewees. These sessions were followed by debriefings. Enumerators were all fluent in English plus at least one of the languages in which the questionnaires had been translated.

**Figure 2:**
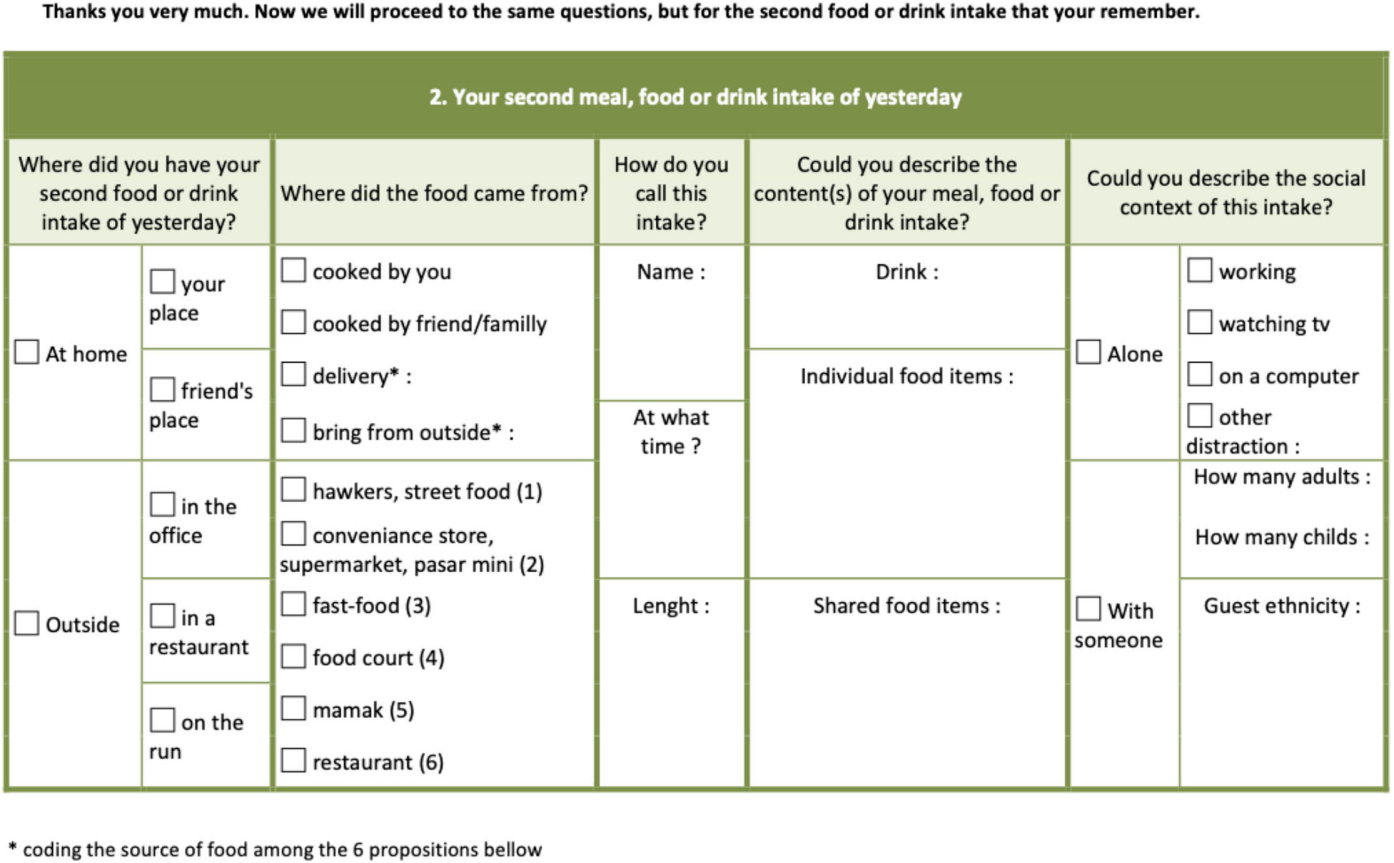
Adapted 24-h recall, food intake recording method.

Particular attention was paid to the use of the food day reconstruction tool: the interviewee's 24-h food intake. The data collection were pitched in the middle ground between a qualitative approach and a quantitative approach. The questions were intended to be a sort of guide to help the interviewee to remember the food intake composition and structure, the time of consumption, the conditions of the acquisition, and the socio-technical contexts of consumption, including the ethnicity of the persons with whom the food was shared. The form and sequence of questions have been developed to reflect the Malaysian context, including the high frequency of consuming food away from home, or as takeaway food, and the large variety in eating places (see Figure 2).

The data collection was conducted on “normal” periods of the year (out of the religious or national feasts) between January and May 2013.

## Already Published Results and Work Perspectives

Some results have been produced and published (2–5, 8, 26, 27). But the interest of the MFB database is far from exhausted. It allows a deep description of food patterns and an analysis of their social determinants like:

- Describe the food habits, practices, social norms, social representations, and beliefs, pertaining to food, food patterns, and temporalities.
- Measure frequency of eating out.
- Identify food lifestyles with a focus on ethnicity.
- Analyze the correlation between lifestyles and above-mentioned characteristics.
- Explore the correlation between lifestyles and BMI.
- Analyze risk perceptions linked to food.

## Conclusion

The MFB database is made available in an “open science” philosophy. Let us remember the spirit of Open Science. It is a movement which consists in opening the research process to all types of actors concerned by the topic (scientific partners, economical actors, public institutions, citizens, NGOs, etc.). It uses the opportunity represented by the “digital mutation” to make research data easily accessible and reusable (28). In our case, the MFB1 report has already been freely accessible since 2015, and the MFB2 will be available shortly. The provision of the database itself is a new step. Sharing research results and data, not only between scientists but also with all stakeholders in society, is a way of disseminating and a way of developing partnership relations with all the different actors concerned by the subject of the research. But it is also a way of advancing knowledge, a way to co-construct the knowledge. And finally, the objective of open science is to accelerate the process of scientific production itself (29, 30).

It is possible to carry out comparative analysis between different time periods in Malaysia and with other countries. It can be used by researchers, public and private decision-makers, educators, and students. Researchers to carry out original work based on secondary data processing, or to make comparisons with other data of the same nature. As part of educational activities, academics and students can do secondary analysis or compare their data collected on subsets of the national population (region, group ethnic or social group) with the database. Public and private decision-makers of the public health sector or food industry and food service sector will find data to support strategical decisions.

## Data Availability Statement

The datasets presented in this study can be found in online repositories. The names of the repository/repositories and accession number(s) can be found in the article/Supplementary Material.

## Ethics Statement

The studies involving human participants were reviewed and approved by Taylor's University Ethical Committee. The patients/participants provided their written informed consent to participate in this study.

## Author Contributions

J-PP and NR obtained the funding. J-PP, LT, CL, EM, and IN designed the study, involved in data collection, and supervision. J-PP, LT, CL, EM, TF, ADa, ADu, AR, YA, and KN contributed to the data analysis. J-PP drafted the manuscript. All authors critically revised the manuscript.

## Funding

The project was made possible with the help of Malaysian and international public and private support. Academics partners: Taylor's University (TUC Chair 2012-3 & TRC2013) 31%, Toulouse University (ISTHIA-TTUC2012-3) 31%, Ministry of Higher Education Malaysia, Long Research Grant Scheme (LRGS) “Social National Cohesion”, (Prof. Shamsul, LRGS/BU/2011/UKM/CMN) 6%, CNRS-France, LIA “Food, Cultures and Health” (LIA-CNRS France-Malaisie2016) 2%. Industrial partners: Observatory of Food Habits of French Dairy Industry (CNIEL-MFB 2012-4) 23%, Coca-Cola-Malaysia 5%, and Nestlé-Malaysia 2%. The funders were not involved in the study design, collection, analysis, interpretation of data, the writing of this article or the decision to submit it for publication.

## Conflict of Interest

The authors declare that the research was conducted in the absence of any commercial or financial relationships that could be construed as a potential conflict of interest.

## Publisher's Note

All claims expressed in this article are solely those of the authors and do not necessarily represent those of their affiliated organizations, or those of the publisher, the editors and the reviewers. Any product that may be evaluated in this article, or claim that may be made by its manufacturer, is not guaranteed or endorsed by the publisher.
