# Supplementary material for: The Malaysian Food Barometer Open Database: An Invitation to Study the Modernization of Malaysian Food Patterns and Its Economic and Health Consequences
Source: Front Nutr. 2022 Jan 19;8:800317. doi: 10.3389/fnut.2021.800317 (PMC8809384; doi:10.3389/fnut.2021.800317)
Supplement: Supplementary file 1 [file Data_Sheet_1.docx]

Presentation of the questionnaire is different from the data collection one.

| Respondent Name |  | | |
| --- | --- | --- | --- |
| Address |  | | |
|  |  | | |
| City |  | Postcode |  |
| Contact no | (O) | (H) | (HP) |
| Email |  | | |
| Date |  | | |
| Time Start |  | Time End |  |
| Interviewer |  | | |

| Z1 North | Code |  | Z2 Central | Code |  | Z3 South | Code |
| --- | --- | --- | --- | --- | --- | --- | --- |
| Perlis | 1 |  | Perak | 4 |  | N,Sembilan | 7 |
| Kedah | 2 |  | Kuala Lumpur | 5 |  | Melaka | 8 |
| Penang | 3 |  | Selangor | 6 |  | Johor | 9 |

| Z4 east Coast | Code |  | Z5 East Malaysia | Code |  | Q6 | Code |
| --- | --- | --- | --- | --- | --- | --- | --- |
| Pahang | 10 |  | Sabah | 13 |  | Non – Malay Bumiputra | 1 |
| Terengganu | 11 |  | Sarawak | 14 |  | Malay | 2 |
| Kelantan | 12 |  | Labuan | 15 |  | Indian | 3 |
|  |  |  |  |  |  | Chinese | 4 |
|  |  |  |  |  |  | Other | 5 |

| Q2.Gender | Code |  | Q3. Living area | Code |
| --- | --- | --- | --- | --- |
| Male | 1 |  | Urban | 1 |
| Female | 2 |  | Rural | 2 |

| **Introduction**  Good morning / afternoon / evening, my name is ____________. We are conducting a survey in the frame of research under the Ministry of Higher Education.  The objective is to study Malaysian food habits, for the purpose of improving public health. This research focuses on people of aged 15 and above.  Full anonymity and confidentiality are guaranteed.  This questionnaire will take approximately 30 – 45 minutes.  早安 ／ 午安 ／ 下午 好，我的名字是 .  我们正为大马教育部进行在进行一项关于大马人饮食习惯与大众健康的调查访问。  我们保证不会泄漏您的资料。我们希望您可以参与这份大约30－45分钟的问卷回答。  *Selamat pagi / tengah hari / petang, nama saya _______. Kami sedang menjalankan kajian sejajar dengan penyelidikan inisiatif Kementerian Pendidikan Tinggi. Objektifnya adalah untuk mengkaji tabiat pemakanan penduduk Malaysia, untuk memperbaikikan taraf kesihatan awam. Kajian ini ditujukan untuk golongan respondent yang berumur 15 tahun dan ke atas. Maklumat yang diberikan ini akan hanya digunakan bagi tujuan kajian sahaja dan maklumat responden adalah sulit.*  *Soal selidik ini akan mengambil masa lebih kurang 30 - 45 minit.* |
| --- |

| **Section A: Filter Questions** |
| --- |

| Q1 | Malaysian citizen  马来西亚公民  *Warganegara Malaysia* | SA | Route |
| --- | --- | --- | --- |
|  | Yes  是  Ya | 1 | Continue |
|  | No  不是  Bukan | 2 | Terminate |

| Q2 | Gender (Ask only if needed)  性别  Jantina | SA | Route |
| --- | --- | --- | --- |
|  | Male  男  Lelaki | 1 | Check Quoa |
|  | Female  女  Perempuan | 2 |  |

| Q3 | Living area  生活地区  *Tempat tinggal* | SA | Route |
| --- | --- | --- | --- |
|  | Urban  城市  *Bandar* | 1 | Check Quota |
|  | Suburban  镇  *Pekan* | 2 |  |
|  | Rural  乡村  *Kampung* | 3 |  |

| Q4b | Where did you grow up?  您在哪里度过您的童年呢？  *Dimanakah anda membesar?* | SA | Route |
| --- | --- | --- | --- |
|  | Urban  城市  *Bandar* |  | Check Quota |
|  | Suburban  镇  *Pekan* | 1 |  |
|  | Rural  乡村  *Kampung* | 2 |  |

| Q5 | Date of Birth and Age  生日日期与岁数  *Tarikh Lahir and Umur* | Code | Route |
| --- | --- | --- | --- |
|  |  | Birthdate | Check Quoa |
|  |  | Age |  |

| Q6 | Ethic group or race (do not propose first)  种族  Bangsa | SA | Route |
| --- | --- | --- | --- |
|  | Non – Malay Bumiputra  非巫族土著  Bumiputra bukan Melayu | 1 | Check Quota |
|  | Malay  巫族  *Melayu* | 2 |  |
|  | Indian  印族  *India* | 3 |  |
|  | Chinese  华族  *Cina* | 4 |  |
|  | Others  其他  *Lain-lain* | 5 |  |

| **Section B : Norms on Food** |
| --- |

| Q7 | Generally speaking, how many meals are you consuming per day?  您通常一天内吃多少餐呢？  *Secara umum, berapa hidangan yang anda makan setiap hari?* | SA | Route |
| --- | --- | --- | --- |
|  | 1 meal per day  1天1餐  *1 hidangan setiap hari* | 1 | Check Quota |
|  | 2 meals per day  1天2餐  *2 hidangan setiap hari* | 2 |  |
|  | 3 meals per day  1天3餐  *3 hidangan setiap hari* | 3 |  |
|  | 4 meals day  1天4餐  *4 hidangan setiap hari* | 4 |  |
|  | More than 4 meals  超过4餐  *Lebih daripada 4 hidangan setiap hari* | 5 |  |

| Q8 | Besides those meals, how often do you have food intakes in between meals (tea break, snacks, etc)?  除了这些正餐以外，您是否还有经常进食呢？（茶点，零食等等）  *Selain daripada hidangan tersebut, apakah kekerapan pengambilan makanan sampingan anda (Minum Petang, snek, etc)* | Code | Route |
| --- | --- | --- | --- |
|  | Never  没有 *Tak Pernah* | 1 | Check Quota |
|  | Once to twice a day  1天1或2次 *Satu hingga dua kali sehari* | 2 |  |
|  | Three to four times a day  1天3至4次  *Tiga hingga empat kali sehari* | 3 |  |
|  | Five to six times a day  1天5至6次  *Lima hingga enam kali sehari* | 4 |  |
|  | More than six times a day  1天超过6次  *Lebih daripada enam kali sehari* | 5 |  |

| Q9 | The following question is about your usual meal organization. Could you please explain what your lunch, dinner and supper usually consists of.  接下来的问题是有关于您用餐单。我将会问一些有关于您的早餐，午餐，晚餐，宵夜和茶点的餐单。  *Soalan yang berikut adalah berhubungan dengan jenis-jenis makanan anda. Boleh anda menjelaskan jenis makanan yang anda untuk makan tengah hari, makan malam dan makan lewat malam.*  ***[SHOW CARD]*** | | | | | |
| --- | --- | --- | --- | --- | --- | --- |
|  | A proper breakfast  完整早餐  *Sarapan pagi yang betul* | A proper Lunch  完整午餐  *Makan tengah hari yang betul* | A proper dinner  完整晚餐  *Makan malam yang betul* | A proper supper  完整宵夜  *Makan*  *lewat malam yang betul* | A snack  茶点  *Snek yang betul* | Tea time  下午茶  *Watuk minum* |
|  |  |  |  |  |  |  |

**Q9 Card**

| 1 | 1 Dish of Rice/Noodles mixed with other food in the plate (incl. Rice Porridge or Instant Noodles) +/- Drink  一碟饭／面和配料 （包括粥与快速面）也许  + / -饮料。  *Satu hidangan nasi / mi yang dicampur dengan hidangan sampingan (Termasuk bubur dan mi segera)* + / - *Minuman* |  |
| --- | --- | --- |
| 2 | 1 Dish of Roti Canai/Thosai/Chapati mixed with curry +/- Drink  一张印度煎饼 /薄煎饼还有咖喱，+ / - 饮料  *Satu hidangan roti canai /Thosai / Chapati dengan kari,* + / - *Minuman* |  |
| 3 | 1 Dish of Rice/Noodles with shared dishes (including Lazy Susan) +/- Drink  叫菜肴吃饭或面+ / - 饮料  *Satu hidangan nasi / mi dengan lauk pauk ,* + / - *Minuman* |  |
| 4 | Sandwich/Burger +/- Drink  三文治 ／汉堡包 + / -饮料  *Sandwic / Burger* + / - *Minuman* |  |
| 5 | Cereals with milk+/- Drink  麦片和牛奶 ／+ / -饮料  *Bijirin dengan susu* + / - minuman |  |
| 6 | Anglo-Saxon Breakfast with Sausage and Eggs +/- Drink  英式早餐，香肠和鸡蛋+ / -饮料  *Sarapan barat dengan sosej dan telu r*+ / - *Minuman* |  |
| 7 | Continental Breakfast with Toasts and Hot +/- Drink  中式早餐，烤面包+/-热饮料  Sarapan timur dengan roti bakar +/-minuman panas |  |
| 8 | Pastries or Shared dishes (Dim Sum) +/- Drink  西式糕点或点心+/-饮料  *Pastri atau Dim Sum +/- minuman* |  |
| 9 | Small items (Nuts, Fruits, Kuih-Muih, etc) +/- Drink  小件食品 （花生，水果，糕点，等等）+/-饮料  *Makanan kecil (kacang,buah-buahan,kuih muih dan lain lain)* |  |
| 11 | 1 Dish of Rice/Noodles mixed with other food in the plate  一碟饭／面和配料+ / -饮料。  *Satu hidangan nasi / mi yang dicampur dengan hidangan sampingan* + / - *minuman* |  |
| 12 | 1 Dish of Roti Canai/Thosai/Chapati mixed with curry  一张印度煎饼 /薄煎饼还有咖喱，+ / - 饮料  *Satu hidangan roti canai /Thosai / Chapati dengan kari,* + / - *Minuman* |  |
| 13 | 1 Dish of Rice/Noodles with shared dishes (including Lazy Susan)  叫菜肴吃饭或面+ / - 饮料  *Satu hidangan nasi / mi dengan lauk pauk ,* + / - *Minuman* |  |
| 14 | Sandwich/Burger/Savory Pastries  三文治 ／ 汉堡包和 ／ 西洋糕点  *Sandwic / Burger dengan kentang* |  |
| 15 | Western Structure (Starter+/- Main Course+/- Dessert)  西餐，前菜 － 主菜 － 甜点  *Hidangan barat (Pembukaan selera, hidagan utama, pencuci mulut)* |  |
| 16 | Small items (Nuts, Fruits, Kuih-Muih, etc)  小件食品 （花生，水果，糕点，等等）+/-饮料  *Makanan kecil (kacang,buah-buahan,kuih muih dan lain lain)* |  |
| 17 | 1 Dish of Rice/Noodles mixed with other food in the plate  一碟饭／面和配料  *Satu hidangan nasi / mi yang dicampur dengan hidangan sampingan* | **17a )** +Fruits/Desserts  + 水果 / 甜点  *+ Buah Buahan / Desert* |
| 18 | 1 Dish of Roti Canai/Thosai/Chapati mixed with curry  一张印度煎饼 /薄煎饼还有咖喱。  *Satu hidangan roti canai /Thosai / Chapati dengan kari* | **18a)**  +Fruits/Desserts  + 水果 / 甜点  *+ Buah Buahan / Desert* |
| 19 | 1 Dish of Rice/Noodles with shared dishes (including Lazy Susan)  叫菜肴吃饭或面  *Satu hidangan nasi / mi dengan lauk pauk* | **19a)** + +Fruits/Desserts  + 水果 / 甜点  *+ Buah Buahan / Desert* |
| 20 | Sandwich/Burger/Savoury Pastries  三文治 ／ 汉堡包和 ／ 西洋糕点  *Sandwic / Burger dengan kentang* | **20a)** +Fruits/Desserts  + 水果 / 甜点  *+ Buah Buahan / Desert* |
| 21 | Western Structure (Starter+/- Main Course+/- Dessert)  西餐，前菜 － 主菜 － 甜点  *Hidangan barat (Pembukaan selera, hidagan utama, pencuci mulut)* | **21a)** Starter+Main Course  前菜 － 主菜  *Hidangan barat (Pembukaan selera dan hidagan utama,*  **21b)** Starter+Dessert  前菜 － 甜点  *Hidangan barat (Pembukaan selera, dan pencuci mulut)* |
| 22 | Small items (Nuts, Fruits, Kuih-Muih, etc)  小件食品 （花生，水果，糕点，等等）+/-饮料  *Makanan kecil (kacang,buah-buahan,kuih muih dan lain lain)* |  |
| 23 | 1 Soup  1 汤  *1 sup* |  |
| 24 | Steamboat  火锅  *Steamboat* |  |
| 25 | Buffet  自由餐  *Buffet* |  |
| 26 | Local/Western Pastries (Kuih Muih, Cakes, Vienoisseries, Sandwich) +Beverage  本地 / 西方糕点 ＋ 饮料  *Kuih Muih / Pastri + minuman* |  |
| 27 | Sweet/Savory Snacks (Biscuits, Chocolates, Candies, Chips, Nuts) + Beverage  甜/酸，咸零食（饼干，巧克力，薯片，花生） + 饮料  *Snek Manis atau masin masam (Biskut,coklat,gula,cips,kaan)+minuman* |  |
| 28 | Rice/Roti Canai/Noodles + Beverage  饭/印度煎饼/面+饮料  *Nasi / Roti Canai / Mi + Minuman* |  |
| 29 | Fruits/Hot or Cold Dessert+ Beverage  水果 / 热或冷的糖水，甜点 ＋ 饮料  *Buah-buahan / pencuci muluh panas atau sejuk + minuman* |  |
| 30 | 1 Beverage  1 杯饮料  *1 minuman* |  |


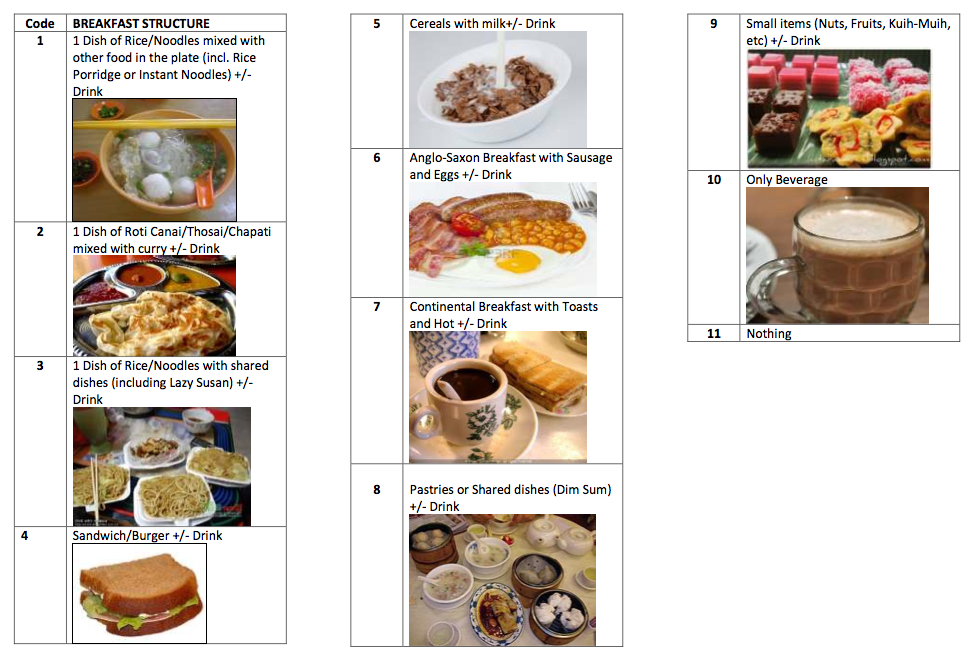


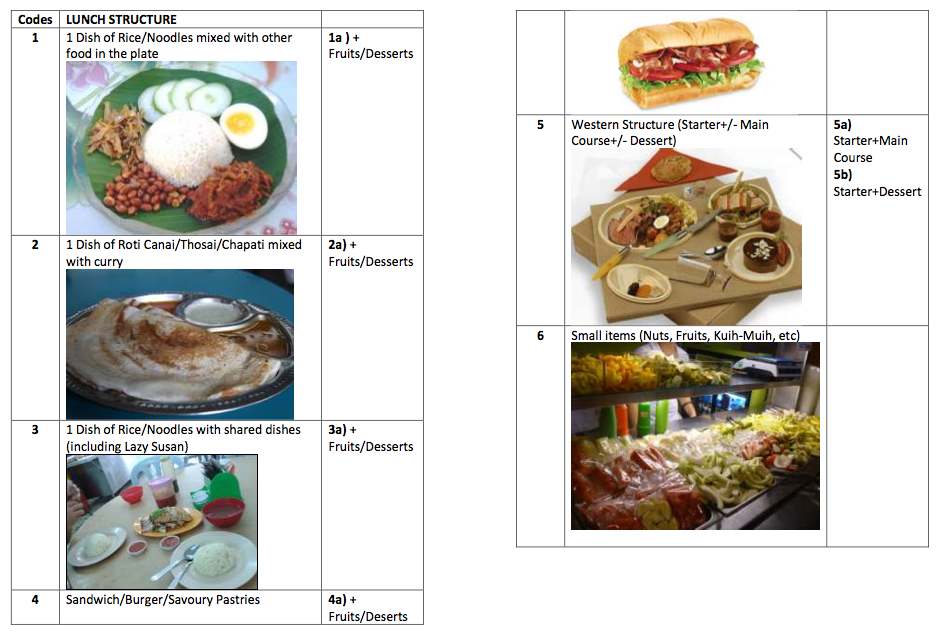


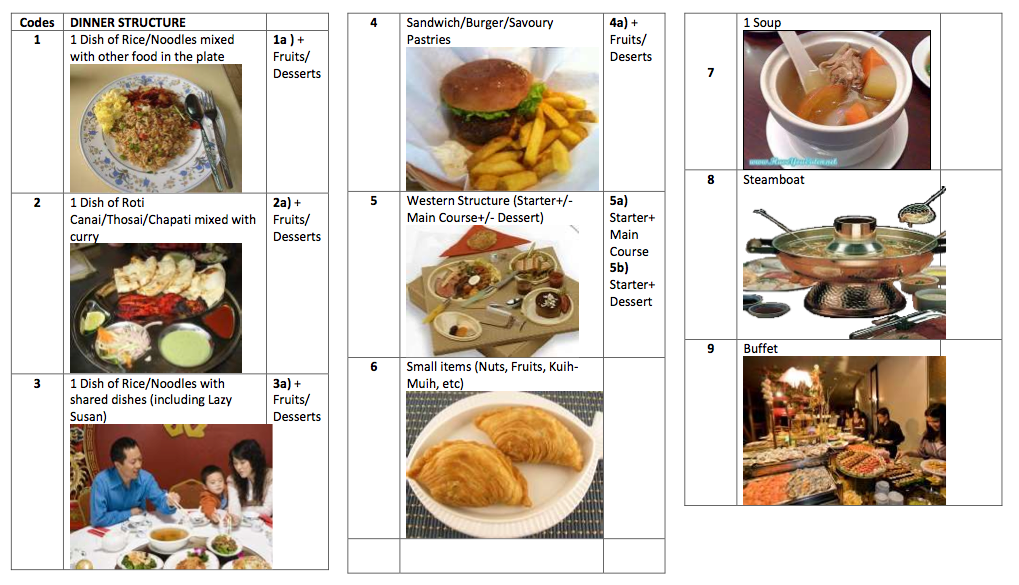


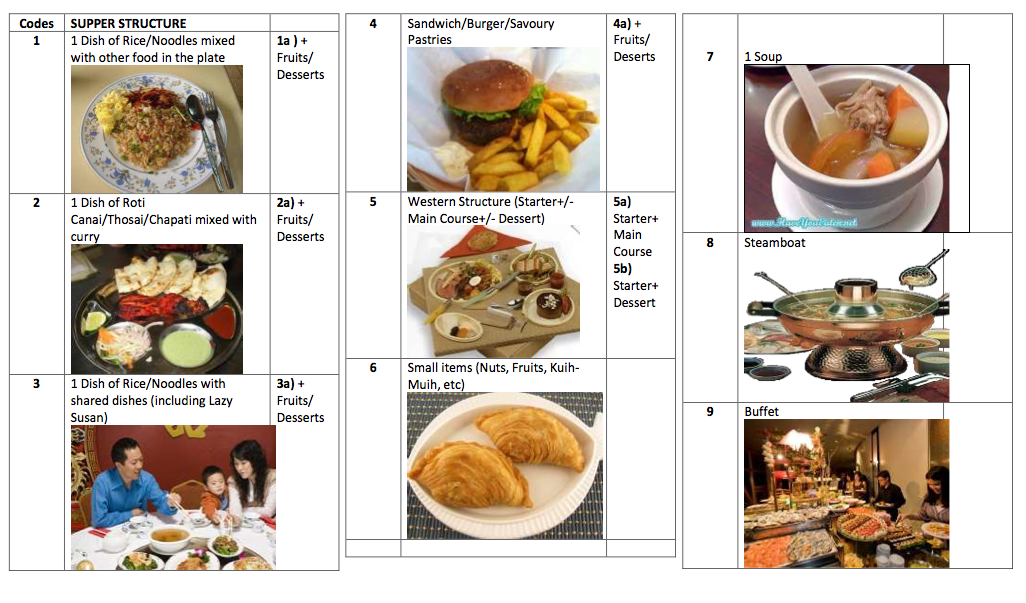


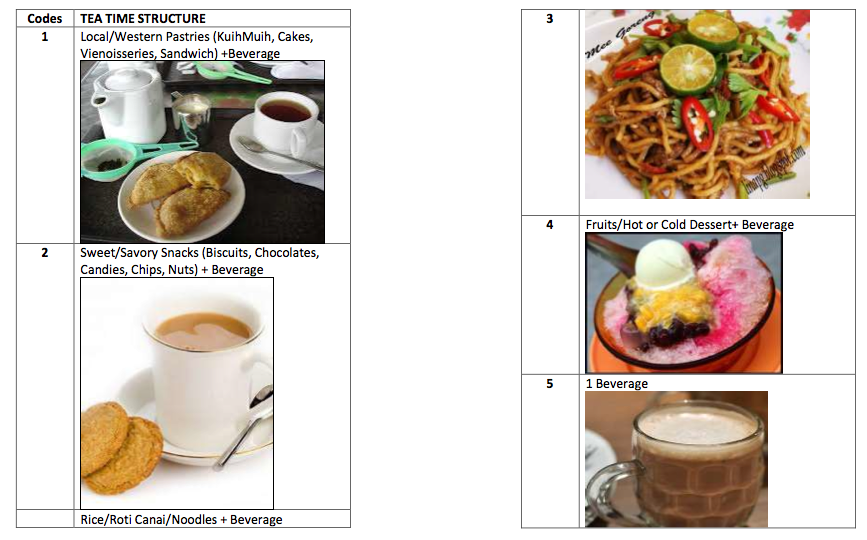


| Q10 | For the following meals, could you tell me which food and drink that you have to include in order to be a proper meal, a meal that you are supposed to eat every day?  您可以告诉我以下的餐点，有那些食物和饮料是必需具备在正餐的，就是您每一天都应该有吃喝的。  *Di antara makanan dan minuman yang berikut, yang mana anda anggap sebagai makanan dan minuman yang boleh menglengkapkan hidangan anda setiap hari?* |
| --- | --- |

|  | Generally speaking,  通常  *Secara umumnya,* | A proper breakfast  完整早餐  *Sarapan pagi yang betul* | A proper Lunch  完整午餐  *Makan tengah hari yang betul* | A proper dinner  完整晚餐  *Makan malam yang betul* | A proper supper  完整宵夜  *Makan*  *lewat malam yang betul* | A snack  茶点  *Snek yang betul* | Tea time  下午茶  *Watuk minum* |
| --- | --- | --- | --- | --- | --- | --- | --- |
|  | SA |  |  |  |  |  |  |
|  | C. Beverage  饮料  *Minuman* |  |  |  |  |  |  |
| 1 | Water  白开水  *Air* | 1 | 1 | 1 | 1 | 1 | 1 |
| 2 | Coffee  咖啡  *Kopi* | 2 | 2 | 2 | 2 | 2 | 2 |
| 3 | Tea  茶  *Teh* | 3 | 3 | 3 | 3 | 3 | 3 |
| 4 | Tea Tarik  奶茶／拉茶  *Teh tarik* | 4 | 4 | 4 | 4 | 4 | 4 |
| 5 | Chocolate Drink  巧克力饮料  *Minuman Coklat* | 5 | 5 | 5 | 5 | 5 | 5 |
| 6 | Cow’s milk  牛奶  *Susu Lembu* | 6 | 6 | 6 | 6 | 6 | 6 |
| 7 | Soy Milk  豆奶  *Susu Soya* | 7 | 7 | 7 | 7 | 7 | 7 |
| 8 | Herbal Tea  凉茶  *Teh Herba* | 8 | 8 | 8 | 8 | 8 | 8 |
| 9 | Fruit Juice  果汁  *Jus Buah-buahan* | 9 | 9 | 9 | 9 | 9 | 9 |
| 10 | Soda  苏打饮料  *Soda* | 10 | 10 | 10 | 10 | 10 | 10 |
| 11 | Cordials  甜饮  *Kordial* | 11 | 11 | 11 | 11 | 11 | 11 |
| 12 | Alcohol  酒精饮料  *Alkohol* | 12 | 12 | 12 | 12 | 12 | 12 |
| 13 | Other  其他  *Lain-lain* | 13 | 13 | 13 | 13 | 13 | 13 |
| 14 | I never have this kind of meal  *Saya tak pernah ambil jenis makanan ini*  我没有吃这样形式的餐点 | 14 | 14 | 14 | 14 | 14 | 14 |

**Q10 Card**

| 1 | Water  白开水  *Air* |
| --- | --- |
| 2 | Coffee  咖啡  *Kopi* |
| 3 | Tea  茶  *Teh* |
| 4 | Tea Tarik  奶茶／拉茶  *Teh Tarik* |
| 5 | Chocolate Drink  巧克力饮料  *Minuman Coklat* |
| 6 | Cow’s milk  牛奶  *Susu Lembu* |
| 7 | Soy Milk  豆奶  *Susu Soya* |
| 8 | Herbal Tea  凉茶  *Teh Herba* |
| 9 | Fruit Juice  果汁  *Jus Buah-buahan* |
| 10 | Soda  苏打饮料  *Soda* |
| 11 | Cordials  甜饮  *Kordial* |
| 12 | Alcohol  酒精饮料  *Alkohol* |
| 13 | Other  其他  *Lain-lain* |
| 14 | I never have this kind of meal  *Saya tak pernah ambil jenis makanan ini*  我没有吃这样形式的餐点 |

| **Section C: Food intakes of the last 24 hours** |
| --- |

| Q11 | Before we proceed to recall your food consumption for yesterday, we will proceed to a recall your food consumption of the previous days, before yesterday. It is not common to remember that we have been eating. However I would like you to try to remember how many meals you had at your place for the last seven days.  在我们还没有继续有关于您昨天所吃过的食物，现在让我们想想您前几天所享用过的食物。  虽然要想回数天前吃过的食物并不简单。但是我希望您可以尽量回想您过去7天里所吃过所有餐点的次数。  *Sebelum kita terus dengan soalan pemakanan anda semalam, kita ingin anda cuba ingat kembali makanan anda hari sebelumnya. Ini adalah perkara yang luar biasa, walau bagaimanapun, saya ingin anda cuba ingat seberapa banyak boleh.* | Code | Route |
| --- | --- | --- | --- |
|  | I ate xx times at home last week  我一个星期前在家里总共吃了xx 餐  *Saya makan xx kali di rumah pada minggu lepas* |  | Continue |
|  | I ate xx times outside last week  我一个星期前在外头总共吃了xx 餐  *Saya makan xx kali di luar pada minggu lepas* |  |  |

[ IF IT CAN FACILITATE THE ANSWER TELL “IF *IT’S EASIER FOR YOU TO REMEMBER HOW MANY MEALS YOU HAD OUTSIDE YOU CAN TELL ME”.]*

| Q12 | Now I would like you to remember what and how you have been eating and drinking for the whole day yesterday from the moment you woke up to the moment you went to sleep.  It doesn’t matter if it wasn’t what you usually eat. It is important to me to understand the context in which you took your food. For this purpose, I will ask you several questions that can help you to remember your different food and drink intake.  现在我希望您可以想想您在昨天所吃喝过的食物和饮料。无论那是不是您平时所享用的食物或饮料。  我非常希望可以详细了解您用餐时的餐单。  所以我将会询问一些可以帮助您回忆有关您所享用过的食物餐单的问题。  *Sekarang saya ingin anda ingat apa dan bagaimana anda telah makan dan minum sepanjang hari semalam.*  *Ia adalah penting bagi saya untuk memahami dan menganalisis persekitaran pemakanan anda.*  *Untuk tujuan ini, saya akan tanya anda beberapa soalan yang boleh membantu anda untuk mengingati pemakanan berbeza* |
| --- | --- |

**Card for 24h Recall – Questionnaire includes seven food intakes (English & Chinese)**

| Your first meal, food or drink intake of yesterday 您昨天所享用的第1餐。 | | | | | | |
| --- | --- | --- | --- | --- | --- | --- |
| Where did you have your first food or drink intake yesterday?  您在哪里享用呢？ | | Where did the food come from?  在哪里购买呢？ |  | Could you describe the content(s) of your meal, food or drink intake?  您可以告诉我您的用餐内容吗？ | Could you describe the social context on this intake?  您可以描述一下这一餐的背景吗？ | |
| 1. At home  在家里 | 3. your place我的家 | 8. cooked by you自己准备的 | Name :  名字 | Qty :  数量 | 18. Alone  自己一人 | 19. With someone 和同伴 |
|  |  | 9. cooked by friend / family  朋友 ／ 家人准备的 |  |  |  |  |
|  | 4. friend’s place  朋友的家 | 10. delivery* : 递送 |  | Brand :  品牌 |  |  |
|  |  | 11. brought from outside* :  外头打包的 |  |  |  |  |
| 2. Outside  外头 | 5. In the office  办公室 | 12. hawkers, street food (1)  小贩，街边摊 | At what time?  时间 | Drinks :  饮料 | If with someone  和同伴 | How many adults :  成人数目 |
|  |  | 13. convenience store, supermarket, pasar mini (2)  便利店，超级市场，迷你市场 |  |  |  | How many children :  小孩数目 |
|  | 6. in a restaurant 餐馆 | 14. fast – food (3) 快餐 |  | Individual food items :  个人点餐项目 |  | Guest (s) ethnicity :  人客种族 |
|  |  | 15. food court (4) 美食广场 |  |  |  |  |
|  | 7. in a hurry忙碌中 | 16. mamak (5) 嘛嘛档口 |  | Shared food items :  共享菜肴项目 | Activities during food intakes  一边用餐一边的活动 | 20. working工作 |
|  |  | 17. restaurant (6) 餐馆 |  |  |  | 21. watching TV看电视 |
| *Coding the source of food among the 6 propositions below | | | | |  | 22. on a computer 电脑 |
|  |  |  |  |  |  | 23.Meal time 用餐时间 |
|  |  |  |  |  |  | 24. other distraction : 其他 |

**Card for 24h Recall – Questionnaire includes seven food intakes (English & Malay)**

| Your first meal, food or drink intake of yesterday / *Pengambilan hidangan pertama anda, makanan atau minuman semalam* | | | | | | |
| --- | --- | --- | --- | --- | --- | --- |
| Where did you have your first food or drink intake yesterday? *Dimanakah anda mengambil makanan pertama atau minuman pertama semalam?* | | Where did the food come from?  *Disediakan oleh siapa?* | Could you describe the content(s) of your meal, food or drink intake?  *Bolehkah anda menerangkan kandungan makanan, atau minuman anda?* | | Could you describe the social context on this intake?  *Bolehkah anda menerangkan situasi anda semasa pengambilan ini?* | |
| 1. At home  *Di Rumah* | 3. your place  *tempat anda* | 8. cooked by you  *dimasak oleh saya* | Name :  *Nama* | Qty :  *Kuantiti* | 18. Alone  *Sendiri* | 19. With someone  *Dengan seseorang* |
|  |  | 9. cooked by friend / family  *dimasak oleh kawan / keluarga* |  |  |  |  |
|  | 4. friend’s place  *tempat Kawan* | 10. delivery* :  *penghantaran* |  | Brand :  *Jenama* |  |  |
|  |  | 11. brought from outside* :  *Bungkus/ Dibawa dari luar* |  |  |  |  |
| 2. Outside  *Di luar* | 5. In the office  *Di dalam pejabat* | 12. hawkers, street food (1)  *Penjaja, makanan tepi jalan* | At what time?  *Pukul berapa?* | Drinks :  *Minuman* | If with someone  *Jika dengan seseorang* | How many adults :  Berapa orang dewasa |
|  |  | 13. convenience store, supermarket, pasar mini (2)  *Kedai serbaneka, pasar raya, pasar mini* |  |  |  | How many children :  *Berapa kanak-kanak* |
|  | 6. in a restaurant  *Di restoran* | 14. fast – food (3) *Makanan segera* |  | Individual food items :  *Barangan makanan individu* |  | Guest (s) ethnicity :  *Etnik tetamu* |
|  |  | 15. food court (4) *food court* |  |  |  |  |
|  | 7. in a hurry  *Dalam kesuntukan masa* | 16. mamak (5)  *mamak* |  | Shared food items :  *Makanan dikongsi* | Activities during food intakes  *Aktiviti-aktiviti semasa pengambilan makanan* | 20. working  *berkerja* |
|  |  | 17. restaurant (6)  *restoran* |  |  |  | 21. watching TV  *Menonton tv* |
| *Coding the source of food among the 6 propositions below | | | | |  | 22. on a computer  *pada komputer* |
|  |  |  |  |  |  | 23.Meal time  *Waktu makan* |
|  |  |  |  |  |  | 24. other distraction :  *Ganguan lain* |

| **Section D: Cooking pratices** |
| --- |

| Q13 | Generally, who is cooking in your household? (Choose one answer)  通常谁为您家人做饭？  *Secara umumnya, siapakah yang masak di rumah anda?* | SA | Route |
| --- | --- | --- | --- |
|  | Myself  我自己  *Sendiri* | 1 | Continue |
|  | My wife  我的妻子  *Isteri Saya* | 2 |  |
|  | My husband  我的丈夫  *Suami Saya* | 3 |  |
|  | My Grandparent(s)  公公／婆婆  *Datuk nenek* | 4 |  |
|  | My Friend  朋友  *Kawan-kawan* | 5 |  |
|  | My mother  我的母亲  *Emak Saya* | 6 |  |
|  | A maid  女佣  *Pembantu rumah* | 7 |  |
|  | I hardly cook at home  我很少在家里做饭  *Saya jarang memasak di rumah* | 8 |  |
|  | Others  其 他 ----------------------------------------  lain-lain | 9 |  |

| Q14 | Which of the following statement do you feel closer to? (Choose one answer)  您认为以下那一项最符合您的想法？  *Antara penyataan berikut yang manakah anda berasa lebih dekat* | SA | Route |
| --- | --- | --- | --- |
|  | Food must be first of all a need  民以食为天  *Makanan adalah satu keperluan* | 1 |  |
|  | Food must be first of all shared with someone  食物是应该分享的  *Makanan sepatutnya dikongsi dengan seseorang* | 2 |  |
|  | Food must be first of all a pleasure  食物是应该被享受的  *Makanan mestilah seronok* | 3 |  |
|  | Food must first of all prevent health problems  食物可以养生  *Makanan pertama sekali mesti dapat mencegah masalah kesihatan* | 4 |  |
|  | Other description  其他 -------------------  *lain-lain* |  |  |

**Q14 Card**

| Food must be first of all a need  民以食为天  *Makanan adalah satu keperluan* | 1 |
| --- | --- |
| Food must be first of all shared with someone  食物是应该分享的  *Makanan sepatutnya mesti dikongsi dengan seseorang* | 2 |
| Food must be first of all a pleasure  食物是应该被享受的  *Makanan mestilah diseronokan* | 3 |
| Food must first of all prevent health problems  食物可以养生  *Makanan pertama sekali mesti dapat mencegah masalah kesihatan* | 4 |

| **Section E: Representations of food** |
| --- |

| Q15 | Could you give me your opinion about halal food products according to the following criteria? **(SHOW CARD)**  您可以告诉我您对清真食物的看法吗？  *Bolehkah anda memberitahu saya mengenai pendapat anda tentang produk makanan halal mengikut criteria berikut?* | | | |
| --- | --- | --- | --- | --- |
|  |  | Yes  认同  *Ya* | No  不认同  *Tidak* | I Don’t know / I don’t care  不清楚 ／无所谓  *Tidak tahu / Tidak peduli* |
| a | I always eat halal products  我时常使用清真食品  *Saya selalu gunakan produk makanan halal* | 1 | 2 | 3 |
| b | I pay attention to halal food  我非常留意该食物使否清真食物  *Saya selalu memberi perhatian kepada makanan halal* | 1 | 2 | 3 |
| c | Halal food tastes better  它的味道比较好吃  *Makanan Halal rasa lebih baik* | 1 | 2 | 3 |
| d | Halal food minimises animal suffering  清真食物过程减少动物的挣扎痛苦  *Makanan Halal kurangkan penderitaan binatang* | 1 | 2 | 3 |
| e | Halal food is more expensive  清真食物价钱比较昂贵  *Lebih mahal* | 1 | 2 | 3 |
| f | Halal food is more hygienic  清真食物比较讲究卫生  *Mempunyai kualiti kebersihan yang lebih baik* | 1 | 2 | 3 |

**Q15 Card**

|  | Yes  认同  *Ya* | No  不认同  *Tidak* |
| --- | --- | --- |
| I always eat halal products  我时常使用清真食品  *Saya selalu gunakan produk makanan halal* | 1 | 2 |
| I pay attention to halal food  我非常留意该食物使否清真食物  *Saya selalu memberi perhatian kepada makanan halal* | 1 | 2 |
| Halal food tastes better  它的味道比较好吃  *Makanan Halal rasa lebih baik* | 1 | 2 |
| Halal food minimises animal suffering  清真食物过程减少动物的挣扎痛苦  *Makanan Halal kurangkan penderitaan binatang* | 1 | 2 |
| Halal food is more expensive  清真食物价钱比较昂贵  *Lebih mahal* | 1 | 2 |
| Halal food is more hygienic  清真食物比较讲究卫生  *Mempunyai kualiti kebersihan yang lebih baik* | 1 | 2 |

| Q16 | I will propose different situations, do the following suggestions differ nowadays to what you’ve been used to do in the past?  我将会根据以下不同的情况，您是否可以让我知道您有改变任何以前的生活习惯吗？  *Saya akan mencadangkan beberapa situasi, adakah cadangan berikut berbeza pada masa sekarang berbanding yang apa yang anda telah lakukan pada masa dahulu?*  ***[Show Card]*** | | | | |
| --- | --- | --- | --- | --- | --- |
|  |  | Did not do it  没有  *Tidak pernah* | less often  很少  Kurang  sekali | same  一样  *Sama sekali* | more often  时常  Sentiasa |
| a | Eating alone  自己单独用餐  *Makan sendiri* | 1 | 2 | 3 | 4 |
| b | Eating in the office/school/workplace  在办公室 ／学校用餐  *Makan di dalam pejabat / Tempat kerja* | 1 | 2 | 3 | 4 |
| c | Eating at casual restaurant  在普通餐馆用餐  *Makan di restoran yang kasual* | 1 | 2 | 3 | 4 |
| d | Eating at food court & fast food  在美食坊或快餐店用餐  *Makan di medan selera & makanan segera* | 1 | 2 | 3 | 4 |
| e | Eating in a mamak restaurant  在嘛吗档用餐  *Makan di restoran mamak* | 1 | 2 | 3 | 4 |
| f | Eating at home  在家里用餐  *Makan di rumah* | 1 | 2 | 3 | 4 |
| g | Inviting someone to your house  邀请朋友到您家做客  *Menjemput seseorang ke rumah saya untuk makan* | 1 | 2 | 3 | 4 |
| h | Being invited at someone’s place  被朋友邀请到他家做客  *Dijemput ke tempat seseorang untuk makan* | 1 | 2 | 3 | 4 |

**Q16 Card**

| Did not do it  没有  *Tidak penah* | less often  很少  Kurang  sekali | same  一样  *Sama sekali* | more often  时常  Sentiasa |
| --- | --- | --- | --- |
| 1 | 2 | 3 | 4 |

| Q17 | Could you tell me 2 dishes that best represent Malaysian food  请您说出2样最能代表马来西亚食物的菜肴  *Boleh beritahu saya 2 hidangan yang terbaik yang mewakili makanan Malaysia?* | MA | Route |
| --- | --- | --- | --- |
|  |  | 1 | Continue |
|  |  | 2 |  |

| Q18 | Could you tell me 2 ingredients/raw materials that best represent Malaysian food?  请您说出2样最能代表马来西亚食物的烹调原料  *Boleh beritahu saya 2 bahan yang terbaik yang mewakili makanan Malaysia?* | MA | Route |
| --- | --- | --- | --- |
|  |  | 1 | Continue |
|  |  | 2 |  |

| Q19 | What does “Eating Well” mean to you?  您认为“吃得好”是什么定义呢？  *Pada pendapat anda, apakah maksud “Pemakan Sempurna” ?*  ***[SHOW CARD]*** | 1^st^  (SA) | 2^nd^  (SA) | Route |
| --- | --- | --- | --- | --- |
|  | Health  吃得健康  *Kesihatan* | 1 | 1 | Continue |
|  | Pleasure  高兴地吃  *Kesukaan* | 2 | 2 |  |
|  | Togetherness  与伙伴大吃大喝  *Keramahtamahan* | 3 | 3 |  |
|  | Fill the stomach  填饱肚子 *Isikan perut* | 4 | 4 |  |
|  | Tradition  吃得传统 *Tradisi* | 5 | 5 |  |
|  | Strength  吃得强壮 *Kuat* | 6 | 6 |  |
|  | Others  其它 *Lain-lain* | 7 | 7 |  |

**Q19 Card**

| Health  吃得健康  *Kesihatan* | 1 |
| --- | --- |
| Pleasure  高兴地吃  *Kesukaan* | 2 |
| Togetherness  与伙伴大吃大喝  *Keramahtamahan* | 3 |
| Fill the stomach  填饱肚子 *Isikan perut* | 4 |
| Tradition  吃得传统 *Tradisi* | 5 |
| Strength  吃得强壮 *Kuat* | 6 |

| **Section F: Health and risk issues** |
| --- |

| Q20 | In your opinion, what are the 2 essential foodstuffs (food items, meals, ingredients, drinks…) that are essentials and beneficial to the health?  在您看来，有哪2种食品对使身体健康来说是不可缺少的  *Pada pendapat anda, apakah 2 bahan makanan yang paling bermanfaat untuk kesihatan?* | MA | Route |
| --- | --- | --- | --- |
|  |  | 1 | Continue |
|  |  | 2 |  |

| Q21 | In your opinion, what are the 2 essential foodstuffs to reduce to be in good health?  在您看来， 又有哪2种食品对使身体健康来说是必须要减少的  *Pada pendapat anda, apakah 2 bahan makanan yang perlu dikurangkan untuk kesihatan?* | MA | Route |
| --- | --- | --- | --- |
|  |  | 1 | Continue |
|  |  | 2 |  |

| Q22 | Which of the following risks scares you the most? Identify your top 3 risks  您最担心以下哪一些风险呢？请说出令您最提心吊胆的3项风险  *Yang manakah berikutnya risiko yang paling menakutkan anda? Namakan 3 tiga risiko yang tinggi*  ***[SHOW CARD]*** | | | |
| --- | --- | --- | --- | --- |
|  |  | Top 1 | Top 2 | Top 3 |
| a | Pesticides on agricultural products  农作物上的农药  *Racun perosak untuk produk pertanian* | 1 | 1 | 1 |
| b | Genetically Modify Organizm (GMO)  基因改造食物  *Organizma terubah suai secara genetik* | 2 | 2 | 2 |
| c | Contamination by pollutant  被污染物所污染  *Pencemaran oleh Pencemar* | 3 | 3 | 3 |
| d | Colouring or preservatives  色素和防腐剂  *Pewarna atau pengawet* | 4 | 4 | 4 |
| e | Germs or bacteria in food  食物里的细菌或病菌  *Kuman atau bacteria dalam makanan* | 5 | 5 | 5 |
| f | Food epidemic (i.e. Bird flu)  通过食物传播的流行性疫病 （禽流感等等）  *Kuman atau bacteria dalam makanan (Bird flu…)* | 6 | 6 | 6 |
| g | Unbalanced diet to fat or too much sugar  不均衡的饮食脂肪或糖分过多  *Diet tidak seimbang terhadap lemak atau terlalu banyak gula* | 7 | 7 | 7 |
| h | Expired food  过期的食物  *Makanan tamat tempoh* | 8 | 8 | 8 |
| i | Food allergens (i.e. nuts….)  食物过敏（对花生敏感）  *Alahan Makanan (Kacang dan lain-lain)* | 9 | 9 | 9 |

| Q23 | Do you pratice any physical activities?  您有参加任何体育活动的习惯吗？  *Adakah anda mengamalkan sebarang aktiviti fizikal?* | SA | Route |
| --- | --- | --- | --- |
|  | Yes 有 Ada | 1 | Go to Q23a |
|  | No 没有 *Tidak* | 2 | Go to Q24 |

| Q23a | Type of activities  活动类型 *Jenis Aktiviti* | Duration per acitivity  活动时间 *Jangka masa untuk setiap aktiviti* | Frequency per week  活动数次 *Kekerapan untuk seminggu* |
| --- | --- | --- | --- |
| 1 |  |  |  |
| 2 |  |  |  |
| 3 |  |  |  |
| 4 |  |  |  |
| 5 |  |  |  |

| Q24 | In the last month, how often have you felt nervous and/or stressed ?  在过去一个月中，您有多常感到紧张和/或焦虑？  *Pada bulan lepas, berapa kerap anda berasa gementar dan/atau tertekan?* | SA | Route |
| --- | --- | --- | --- |
|  | Never  从来没有  *Tidak pernah* | 0 | Continue |
|  | Almost never  很少  *Jarang* | 1 |  |
|  | Sometimes  有时候  *Kadang kala* | 2 |  |
|  | Fairly often  很多时候  *Agak Sering* | 3 |  |
|  | Very often  常常  *Sering* | 4 |  |

**Q24 Card**

| Never  从来没有  *Tidak pernah* |
| --- |
| Almost never  很少  *Jarang* |
| Sometimes  有时候  *Kadang kala* |
| Fairly often  很多时候  *Agak Sering* |
| Very often  常常  *Sering* |

| **Section G: Ethnicity indicators** |
| --- |

| Q25a | Please tell us how you define your cultural identity. (3 words)  请告诉我，您如何定义您本身的文化。（3句）  *Sila beritahu kami bagaimana anda menentukan identiti budaya anda? (3 perkataan)* |
| --- | --- |
| No |  |
| 1 |  |
| 2 |  |
| 3 |  |

| Q25b | Please rank 3 best word from below that how you defined your identity in Malaysia?  请从以下的字句排出1－3最能代表您在马来西亚的身份  Sila aturkan perkataan dari 1-3 yang paling terbaik menggambarkan identity anda di Malaysia. |
| --- | --- |


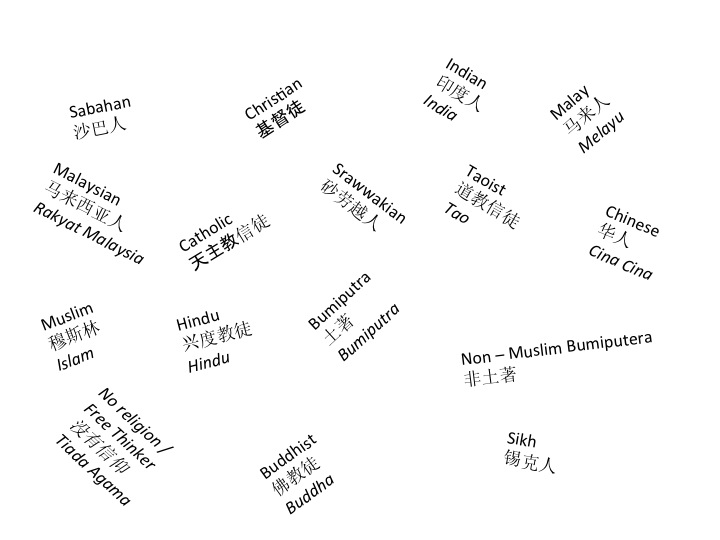


| Q26 | Could you indicate the ethnicity race of both your parents and grandparents in the following chart?  您能在下面的图表中指出您的父母及祖父母的种族吗？  *Bolehkah anda menunjukkan etnik ibu bapa anda dan datuk nenek dalam carta berikut* |
| --- | --- |

| Q27 | Could you indicate the ethnicity / race of your spouse and the family of your spouse?  您能在下面的图表中指出您同伴的父母及祖父母的种族吗？  Bagaimana dengan Suami / isiteri anad? |
| --- | --- |

| Q28 | What languages or dialects do you speak  您通常都用哪一种语言和方言交谈呢？  *Apakah bahasa atau dialek anda ?* | MA | Route |
| --- | --- | --- | --- |
|  | Malay  马来语  *Bahasa Melayu* | 1 | Continue |
|  | English  英语  *Bahasa Inggeris* | 2 |  |
|  | Arabic  阿拉伯语  *Bahasa Arab* | 3 |  |
|  | Mandarin  华语  *Bahasa Cina* | 4 |  |
|  | Cantonese  广东话  *Bahasa Kantonis* | 5 |  |
|  | Hindi  印度话  *Bahasa Hindi* | 6 |  |
|  | Tamil  淡米尔语  *Bahasa Tamil* | 7 |  |
|  | Other:  其他  *Lain-lain* | 8 |  |

| Q29a | What is your religion  您的宗教信仰  *Apakah agama anda* | SA | Route |
| --- | --- | --- | --- |
|  | Muslim  穆斯林  *Islam* | 1 | Continue |
|  | Hindu  兴度教  *Hindu* | 2 |  |
|  | Buddhist  佛教  *Buddha* | 3 |  |
|  | Christian  基督教  *Kristian* | 4 |  |
|  | Taoism  道教  *Tao* | 5 |  |
|  | Other:  其他  *Lain-lain* | 6 |  |
|  | No religion  没有信仰  *Tiada agama* | 7 |  |
|  | Decline to answer (Do not read)  拒绝  *Enggan menjawab* | 8 |  |

[ONLY for Non-Muslim] 非马来穆斯林而已。

| Q29b | Is it a conversion?  您有曾经转换信仰吗？ | SA | Route |
| --- | --- | --- | --- |
|  | Yes 有 | 1 | Go to Q29c |
|  | No 没有 | 2 | Go to 30 |

| Q29c | If YES, you are converted from which religion to religion now?  如果有的话，那您是从什么信仰转换至现在的信仰呢？ |  |  |
| --- | --- | --- | --- |
|  |  | Last time  曾经 | Go to 30 |
|  |  | Now  现在 |  |

| Q30 | Would you describe yourself as  您认为您是  *Anda adalah seorang yang:* | SA | Route |
| --- | --- | --- | --- |
|  | Very religious  非常虔诚  *Sangat beragama* | 1 | Continue |
|  | Moderately religious  适度的虔诚  *Sederhana beragama* | 2 |  |
|  | Lightly religious  有一点虔诚  *Agak beragama* | 3 |  |
|  | Not religious at all  完全不虔诚  *Tidak beragama* | 4 |  |
|  | Decline to answer (Do not read)  拒绝  *Enggan menjawab* | 5 |  |

| **Section H :Socio – Demographic indicators** |
| --- |

| Q31 | How tall are you without your shoes on?  您没有穿鞋的高度是？  *Apakah ketinggian anda tanpa memakai kasut?* | SA | Route |
| --- | --- | --- | --- |
|  |  | cm | Continue |

| Q32 | How much do you weigh without your shoes on?  您没有穿鞋的体重是？  *Berapakah berat badan anda tanpa memakai kasut?* | SA | Route |
| --- | --- | --- | --- |
|  |  | kg | Continue |

| Q33 | What is your occupation?  您的职业是？  *Apakah perkerjaan anda?* | 1st | 2nd | Route |
| --- | --- | --- | --- | --- |
|  | Answer: |  |  | Continue |
|  | Legislator, senior official or manager  立法会议员, 高级职员和经理  *Ahli majlis undangan,pengawa-pengawai kanan, pengurus* | 1 | 1 |  |
|  | Professional  专业人士  *Ahli profesional* | 2 | 2 |  |
|  | Technician and allied professional  技术人员  *Juruteknik dan rakan kongsi profesional* | 3 | 3 |  |
|  | Clerical  文员  *Kerani* | 4 | 4 |  |
|  | Service, shop attendant, or sales person  服务业，店面的服务员，销售人员  *Perkidmatan, pembantu kedai ,jurujual* | 5 | 5 |  |
|  | Skilled worker in agriculture and fisheries  农夫和渔夫  *Pekerja berkemahiran dalam bidang pertanian dan perikanan* | 6 | 6 |  |
|  | Craft and related trades worker  工艺及相关行业的工人  *Kraft dan perdagangan pekerja yang berkaitan* | 7 | 7 |  |
|  | Plant and machine operator or assembler  机台及机器操作员及装配工人  *Loji dan mesin operator serta pemasang* | 8 | 8 |  |
|  | Manual worker  劳工  *Pekerja am* | 9 | 9 |  |
|  | Army personal  军人  *Anggota tentera* | 10 | 10 |  |
|  | Retired  退休者  *Bersara* | 11 | 11 |  |
|  | Student  学生  *Pelajar* | 12 | 12 |  |
|  | Housewife  家庭主妇  *Suri rumah tangga* | 13 | 13 |  |
|  | Unemployed  失业人士  *Penganggur* | 14 | 14 |  |
|  | Others  其他  *Lain-lain* | 15 | 15 |  |
|  | Decline to answer  拒绝  *Enggan menjawab* | 16 | 16 |  |

| Q34 | What is your father’s occupation?  您的父亲是从事哪一个行业  Bagaimana dengan Bapa anda? | SA | Route |
| --- | --- | --- | --- |
|  | Answer : |  | Continue |
|  | Legislator, senior official or manager  立法会议员, 高级职员和经理  *Ahli majlis undangan,pengawa-pengawai kanan, pengurus* | 1 |  |
|  | Professional  专业人士  *Ahli profesional* | 2 |  |
|  | Technician and allied professional  技术人员  *Juruteknik dan rakan kongsi profesional* | 3 |  |
|  | Clerical  文员  *Kerani* | 4 |  |
|  | Service, shop attendant, or sales person  服务业，店面的服务员，销售人员  *Perkidmatan, pembantu kedai ,jurujual* | 5 |  |
|  | Skilled worker in agriculture and fisheries  农夫和渔夫  *Pekerja berkemahiran dalam bidang pertanian dan perikanan* | 6 |  |
|  | Craft and related trades worker  工艺及相关行业的工人  *Kraft dan perdagangan pekerja yang berkaitan* | 7 |  |
|  | Plant and machine operator or assembler  机台及机器操作员及装配工人  *Loji dan mesin operator serta pemasang* | 8 |  |
|  | Manual worker  劳工  *Pekerja am* | 9 |  |
|  | Army personal  军人  *Anggota tentera* | 10 |  |
|  | Retired  退休者  *Bersara* | 11 |  |
|  | Student  学生  *Pelajar* | 12 |  |
|  | Housewife  家庭主妇  *Suri rumah tangga* | 13 |  |
|  | Unemployed  失业人士  *Penganggur* | 14 |  |
|  | Others  其他  *Lain-lain* | 15 |  |
|  | Decline to answer  拒绝  *Enggan menjawab* | 16 |  |

| Q35 | What is the highest level of education that you have completed?  您所完成的最高学历？  *Apakah tahap pendidikan tertinggi yang anda telah lengkapkan?* | SA | Route |
| --- | --- | --- | --- |
|  | No formal education  没有受过正统的教育  *Tiada pendidikan rasmi* | 1 | Continue |
|  | Primary school  小学  *Sekolah rendah* | 2 |  |
|  | Lower secondary school  初中  *Sekolah Menengah Pengajian tinggi* | 3 |  |
|  | Upper secondary school  高中  *Sekolah Menengah Pengajian tinggi* | 4 |  |
|  | Marticulation / Form 6  中六  *Matrikulasi / Form 6* | 5 |  |
|  | College / University  学院 ／ 大学  *Kolaj / Universiti* | 6 |  |
|  | Other:  其他  *Lain-lain* | 7 |  |
|  | Decline to answer  拒绝  *Enggan menjawab* | 8 |  |

| Q36 | What is your marital status?  您的婚姻状况  *Apakah status perkahwinan anda?* | SA | Route |
| --- | --- | --- | --- |
|  | Single or never married  单身或没有结婚  *Bujang atau tidak pernah berkahwin* | 1 | Continue |
|  | Married in monogamous marriage  一夫一妻制的婚姻  *Berkahwin dalam perkahwinan monogami* | 2 |  |
|  | Married in polygamous marriage  一夫多妻制的婚姻  *Berkahwin dalam perkahwinan poligami* | 3 |  |
|  | Living in as married  同居  *Tinggal sebagai berkahwin* | 4 |  |
|  | Widowed  鳏夫 ／ 寡妇  *Janda/duda* | 5 |  |
|  | Separated or married but separated  分居  *Berasing atau berkahwin tapi tinggal berasingan* | 6 |  |
|  | Not living with legal spouse  结婚了，没有与合法伴侣住在一起  *Tidak tinggal dengan pasangan yang sah* | 7 |  |
|  | Divorced  离婚  *Bercerai* | 8 |  |
|  | Decline to answer  拒绝  *Enggan menjawab* | 9 |  |

| Q37 | How many children do you have  您又多少个小孩？  *Berapa kanak-kanak anda mempunyai?* | SA | Route |
| --- | --- | --- | --- |
|  | No children  没有  *Tiada kanak-kanak* | 1 | Continue |
|  | 1 – 2 children  1 - 2个小孩  *1 – 2 orang* | 2 |  |
|  | 3 – 4 children  3 - 4个小孩  *3 – 4 orang* | 3 |  |
|  | 5 children or more  5个小孩或更多  *5 kanak-kanak atau lebih* | 4 |  |

| Q38 | How many family members do you live together with? Including you.  包括自己，您与多少个家人成员生活在一起？  *Berapak ahli keluarga sedang tinggal bersama dengan anda? Termasuk diri sendiri* | SA | Route |
| --- | --- | --- | --- |
|  | 2 – 4 family members  2 - 4 个成员  *2 – 4 ahli keluarga* | 1 | Continue |
|  | 5 – 6 family members  5 - 6 个成员  *5 – 6 ahli keluarga* | 2 |  |
|  | 7 – 8 family members  7 － 8 个成员  *7 – 8 ahli keluarga* | 3 |  |
|  | 9 – 10 family members  9 - 10 个成员  *9 – 10 ahli keluarga* | 4 |  |
|  | More than 10 family members  超过10个成员  *Lebih 10 orang ahli keluarga* | 5 |  |

| Q39 | We would like to know your average monthly household income by using this scale, based on your wages, salaries, pensions, dividends and other income before taxes and other education. Just give the letter of the group your households falls into.(MHI)  这里是一些家庭月收入的组别。我们只想知道您的家庭平均总收入是属于哪一组，请算算所有家人的工资，薪金，退休金，红利支付和其他收入。在所有的税收前的您的家庭收入是多少。  *Kami ingin mengetahui secara puratanya, upah bulanan anda dengan mengunakan skala yang berikut, mengambilkira upah, gaji, pencen, dividen dan pendapatan lain sebelum cukai dan pendidikan yang lain. Anda hanya perlu memberi angka kumpulan isi rumah anda.* | SA | Route |
| --- | --- | --- | --- |
|  | Less than < RM 2,000 | 1 | Continue |
|  | RM 2,001 – RM 5,000 | 2 |  |
|  | RM 5,001 – RM 11,000 | 3 |  |
|  | More than > RM 11,000 | 4 |  |
|  | Decline to answer  拒绝回答  *Enggan menjawab* | 5 |  |

| Q40 | In the past 5 years, would you say that your income (personal income)  在过去的五年里，您会认为您的收入是？  *Dalam 5 tahun yang lalu, anda akan mengatakan bahawa pendapatan anda* | SA | Route |
| --- | --- | --- | --- |
|  | Has decreased  减少  *Telah berkurang* | 1 | Continue |
|  | Has remained stable  一样  *Telah berkurang* | 2 |  |
|  | Has increased  增加  *Telah meningkat* | 3 |  |

| Q41 | We would like to contact you for research purpose in the future. Do you give permission to this?  您允许如果将来有需要，我们可以再联络您吗？  *Kami ingin menghubungi anda untuk tujuan penyelidikan pada masa hadapan. Adakah anda memberi kebenaran?* | SA | Route |
| --- | --- | --- | --- |
|  | Yes  愿意  Ya | 1 | END |
|  | No  不愿意  Tidak | 2 |  |

| Thank You and Close |
| --- |
